# The Influence of Process Parameters on Hydrogen-Terminated Diamond and the Enhancement of Carrier Mobility

**DOI:** 10.3390/ma18010112

**Published:** 2024-12-30

**Authors:** Xingqiao Chen, Mingyang Yang, Yuanyuan Mu, Chengye Yang, Zhenglin Jia, Chaoping Liu, He Li, Nan Jiang, Kazuhito Nishimura, Liangchao Guo, Kuan W. A. Chee, Qilong Yuan, Xiaocheng Li, Hui Song

**Affiliations:** 1 Jiangxi Provincial Key Laboratory of Power Batteries & Energy Storage Materials, Faculty of Materials Metallurgy and Chemistry, Jiangxi University of Sciences and Technology, Ganzhou 341000, China; chenxingqiao@nimte.ac.cn; 2 Key Laboratory of Advanced Marine Materials, Ningbo Institute of Materials Technology and Engineering, Chinese Academy of Sciences, Ningbo 315201, China; yangmingyang@nimte.ac.cn (M.Y.); muyuanyuan@nimte.ac.cn (Y.M.); yangcehngye@nimte.ac.cn (C.Y.); jiazhenglin@nimte.ac.cn (Z.J.); liuchaoping@nimte.ac.cn (C.L.); lihe@nimte.ac.cn (H.L.); jiangnan@nimte.ac.cn (N.J.); kazuhitonishimura@nimte.ac.cn (K.N.); 3 Center of Materials Science and Optoelectronics Engineering, University of Chinese Academy of Sciences, Beijing 100049, China; 4 College of Mechanical Engineering, Yangzhou University, Yangzhou 225127, China; glch2021@yzu.edu.cn; 5 National Laboratory for Physical Sciences at Microscale, University of Science and Technology of China, Hefei 230026, China; kuan.chee@cantab.net

**Keywords:** hydrogen-terminated diamond, surface transfer doping, Hall effect, CH_4_ concentration

## Abstract

With the development of diamond technology, its application in the field of electronics has become a new research hotspot. Hydrogen-terminated diamond has the electrical properties of P-type conduction due to the formation of two-dimensional hole gas (2DHG) on its surface. However, due to various scattering mechanisms on the surface, its carrier mobility is limited to 50–200 cm^2^/(Vs). In this paper, the effects of process parameters (temperature, CH_4_ concentration, time) on the electrical properties of hydrogen-terminated diamond were studied by microwave plasma chemical vapor deposition (CVD) technology, and hydrogen-terminated diamond with a high carrier mobility was obtained. The results show that homoepitaxial growth of a diamond film on a diamond substrate can improve the carrier mobility. Hydrogen-terminated diamond with a high carrier mobility and low sheet resistance can be obtained by homoepitaxial growth of a high-quality diamond film on a diamond substrate with 4% CH_4_ concentration and hydrogen plasma treatment at 900 ℃ for 30 min. When the carrier concentration is 2.03 × 10^12^/cm^2^, the carrier mobility is 395 cm^2^/(Vs), and the sheet resistance is 7.82 kΩ/square, which greatly improves the electrical properties of hydrogen-terminated diamond. It can enhance the transmission characteristics of carriers in the conductive channel, and is expected to become a potential material for application in devices, providing a material choice for its application in the field of semiconductor devices.

## 1. Introduction

As a new type of ultra-wide band gap semiconductor material with a band gap of 5.5 eV, diamond has a series of excellent physical and chemical properties, such as a high breakdown electric field (>1 MV/mm), high intrinsic carrier mobility (highest electron mobility 4500 cm^2^/(Vs) and highest hole mobility 3800 cm^2^/(Vs)), high thermal conductivity (2200 W/(mK) at room temperature), high carrier saturation rate, and low dielectric constant. It is considered to be an ideal material for manufacturing high-power, high-frequency, high-voltage, and high-temperature electronic devices [1,2,3].

Interestingly, by transferring the doping effect and hydrogenating the diamond surface [4,5], a high concentration of two-site hole gas with a low activation energy can be formed on the diamond surface [6], making it exhibit negative electron affinity and surface P-type conductivity [7]. It is a research hotspot in the field of diamond semiconductor devices to use the two-dimensional hole gas on the surface of hydrogen-terminated diamond as the conductive channel of electronic devices [8]. However, due to various scattering mechanisms on the diamond surface, such as surface roughness scattering, phonon scattering, impurity scattering, etc. [9,10], the surface carrier mobility of hydrogen-terminated diamond is limited, being usually below 200 cm^2^/(Vs) [9,10,11]. The electrical properties of hydrogen-terminated diamond directly affect its application in the field of electronic devices. Some studies have found that the carrier density in the conductive channel can be improved by changing the adsorbate. M Kubovic et al. [12,13] systematically studied the effect of adsorbed gas NO_2_ on the conductivity of hydrogen-terminated diamond. The results show that NO_2_ gas can significantly increase the hole density in the conductive channel of the hydrogen-terminated diamond surface. T Wade et al. [14] reported that in the surface conductive channel the carrier transport behavior can be controlled by the surface roughness. An enhanced carrier density can be obtained by fabricating rough hydrogen-terminated diamond surfaces on a microscale. Therefore, the diamond surface will provide more active sites to induce holes. For the mobility, it will be limited by the surface roughness scattering. When the increase in carrier density is greater than the decrease in carrier mobility, the surface conductivity will be improved. Generally, the carrier mobility can be improved by optimizing the surface flatness of the diamond crystal and reducing the impurity content in the diamond crystal [15].

At present, the main method to obtain hydrogen-terminated diamond is to hydrogenate on a high temperature and high pressure (HTHP) [16] or to chemical vapor deposition (CVD) single-crystal diamond substrates. However, the HTHP method will introduce more catalyst impurities, so the effect of hydrogenation is not ideal. The microwave energy used in the preparation process of the MPCVD method is pollution-free, the gas raw material is more pure, and there is no catalyst and impurity incorporation [17,18,19], which can further improve the quality of hydrogen-terminated diamond [20,21]. Although the electrical properties of hydrogen-terminated diamond are related to many factors, they are closely related to the quality of the diamond, and the growth process of the diamond directly affects the quality of the diamond. Therefore, it is of practical significance to study the influence of process parameters on hydrogen-terminated diamond [22]. In this paper, by exploring the effects of hydrogen plasma treatment temperature, time, and different CH_4_ concentrations’ epitaxial growth on hydrogen-terminated diamond, a layer of high-quality diamond film is homoepitaxially grown on a single-crystal diamond substrate, which can avoid the influence of impurity elements on the electrical properties of the material surface, can obtain a smooth and flat surface, avoiding the mechanical damage caused by the secondary processing of the surface to the material surface, and can be used as a substrate material for electronic devices. By studying the above influencing factors, a hydrogen-terminated diamond film with a smooth surface was prepared. The high carrier mobility was 395 cm^2^/(Vs), the sheet resistance was 7.82 kΩ/square, and the surface roughness was below 2 nm.

## 2. Experiments and Methods

The diamond used in this experiment is a CVD single-crystal diamond substrate with a size of 5 mm × 5 mm × 0.5 mm and a crystal plane of (100). All diamond substrates were first acid-washed with piranha solution (volume ratio H_2_O_2_:H_2_SO_4_ = 3:7) for 4 h, then ultrasonically washed with deionized water for 15 min, then ultrasonically treated with anhydrous ethanol for 15 min, and finally ultrasonically washed with deionized water for later use.

(1) The sample was placed in the chamber and vacuumed to 0.5 Pa. Hydrogen was introduced to excite the microwave plasma, and the pressure and power were gradually increased to make the surface of the sample reach about 900 °C. Hydrogen plasma pre-etching was carried out for 10 min. In the process of homoepitaxial growth, on the one hand, the pre-etching process can remove most of the impurities on the surface, such as dust and the non-diamond phase, and improve the purity of the epitaxial layer. On the other hand, it can remove the stress damage caused by the surface polishing process to a certain extent, reduce the defect density of the epitaxial layer, and improve the crystal quality. Then, the homoepitaxial growth of the diamond was carried out by introducing different CH_4_ contents. The temperature was controlled at 950 °C–980 °C, and the growth was carried out at this temperature environment for 8h. Then, the CH4 was turned off and the hydrogen etching was carried out at 900 °C for 30 min. The homoepitaxial growth parameters of high-quality diamond layers are shown in Table 1.
(2) Under the same conditions, 3% CH_4_ was selected for temperature-controlled growth, and the temperature was controlled at 950 °C–980 °C. After 8 h of growth, a high-purity single-crystal diamond homoepitaxial layer was grown on the substrate surface. After CH_4_ was turned off, hydrogen etching was carried out at 900 °C for 15 min, 30 min, and 45 min, respectively.
(3) The samples were placed in a 6 kW microwave plasma chemical vapor deposition (MPCVD) chamber and vacuumed to 0.5 Pa. Then, hydrogen was introduced to excite the plasma, and the power and pressure of the equipment were gradually increased. Hydrogen etching was performed at 600 °C, 700 °C, 800 °C, 900 °C, and 1000 °C, respectively. After the surface temperature of the sample reached the set temperature, the hydrogen etching was maintained at this temperature for 30 min. The temperature parameters of the hydrogen plasma treatment are shown in Table 2.

The crystal quality of the synthesized hydrogen-terminated diamond was characterized by Raman spectroscopy (Labramhr Evolution, Hariba, Japan). The surface roughness and morphology of hydrogen-terminated diamond were characterized by atomic force microscopy (Dimension lcon, Bruker, Billerica, MA, USA). The chemical composition of the hydrogen-terminated diamond surface was characterized by X-ray photoelectron spectroscopy (AXIS SUPRA, Kratos, England). The standard C 1s peak at 284.8 eV was used for spectral calibration, and the chemical bonding on its surface was analyzed. The electrical properties of carrier type, carrier concentration, mobility, and surface conductivity were tested and analyzed by the Hall test system (8404, Lake Shore, Westerville, OH, USA).

## 3. Results and Discussion

**Figure 1:**
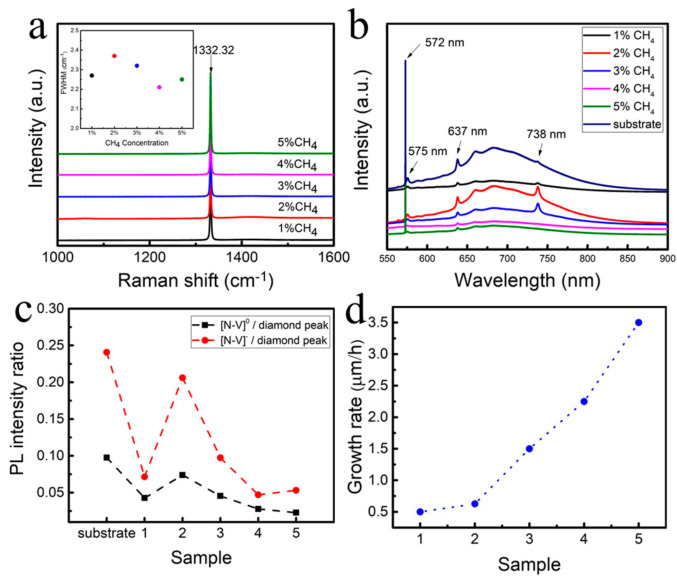
(**a**) Raman spectra of hydrogen-terminated diamonds epitaxially grown at different CH_4_ concentrations; (**b**) photoluminescence spectra of hydrogen-terminated diamonds and diamond substrates epitaxially grown at different CH_4_ concentrations; (**c**) the ratio of [N-V]^0^ and [N-V]^−^ to the pl intensity of the diamond characteristic peak; and (**d**) homoepitaxial growth rate of the different samples.

Figure 1a shows the Raman spectra of hydrogen-terminated diamonds prepared under different CH_4_ concentration atmospheres. It can be analyzed from the diagram that the Raman peaks of hydrogen-terminated diamonds prepared under different conditions are all located at 1332.32 cm^−1^, which is not much different from the standard Raman peak of diamonds, which is 1332.5 cm^−1^, indicating that the internal stress of hydrogen-terminated diamonds prepared under different conditions is a small and tensile stress. The FWHM is less than 2.4 cm^−1^, and the curve in the non-peak region is very smooth, indicating that the prepared hydrogen-terminated diamond has a high surface purity, high crystallinity, and no obvious nitrogen peak, indicating a low nitrogen content. At 4% CH_4_, the FWHM is 2.21 cm^−1^, which is the smallest, because the smaller the full width at half-maximum (FWHM), the less the crystal defects of the diamond, the better the lattice, and the higher the crystallinity. Compared with the hydrogen-terminated diamond prepared by other CH_4_ contents, its half-peak width is the smallest, so its quality is the best [1,3]. Figure 1b shows the photoluminescence spectrum of the diamond substrate and diamond with different hydrogen terminals. It can be seen from the figure that the diamond substrate has obvious [N-V]^0^ and [N-V]^−^ peaks, which are the characteristic peaks of intrinsic diamond at 572 nm. In addition, the peaks at 575 nm and 637 nm correspond to impurities [N-V]^0^ and [N-V]^−^, respectively. The broadband between 600 and 800 nm is considered to be related to the fluorescence of N-related impurities, with a peak at 738 nm. With the homogeneous epitaxial growth of diamond films with different CH_4_ concentrations, the epitaxial layer was gradually purified, the curve tended to be straight, the surface purity was improved, and the intensity peaks of [N-V]^0^ and [N-V]^−^ were weakened. Figure 1c shows the ratio of the characteristic peaks of [N-V]^0^ and [N-V]^−^ to the characteristic peaks of intrinsic diamond, which can show the relative intensity of [N-V]. On the diamond substrate, the relative strength of [N-V]^0^ and [N-V]^−^ is the highest [8]. With the preparation of diamond films at different CH_4_ concentrations, the strength decreases and reaches the lowest at 4% CH_4_ concentration. This is because the process of depositing diamond film gradually reduces the influence of [N-V]^0^ and [N-V]^−^, indicating that the quality of hydrogen-terminated diamond prepared under this condition is better [23]. Figure 1d and Table 3 show the growth rate and deposition thickness of the epitaxial layer of different samples at different CH_4_ concentrations. It can be seen from the chart that when the CH_4_ concentration is less than 3%, the growth rate of the epitaxial layer is lower. This is because the growth effect of diamond is less than the etching effect. When the CH_4_ concentration is higher than 3%, the growth effect is greater than the etching effect, and the growth rate is greatly improved. However, when the CH_4_ concentration is too high, excessive growth will occur, resulting in a decrease in the quality of the epitaxial layer [22,24,25].

**Figure 2:**
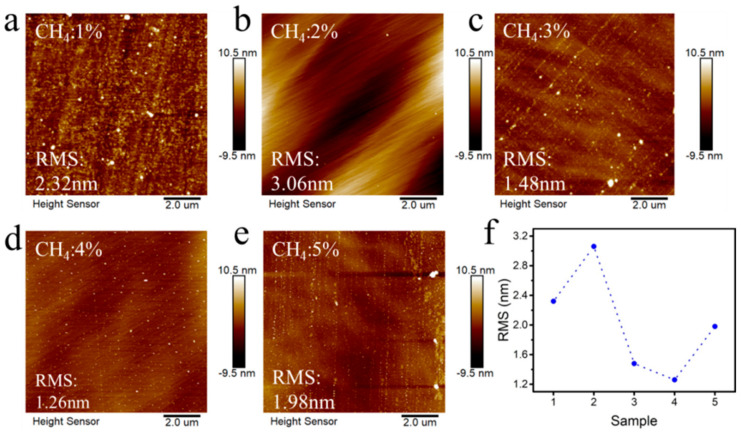
Surface roughness of hydrogen-terminated diamonds epitaxially grown at different CH_4_ concentrations: (**a**) 1%; (**b**) 2%; (**c**) 3%; (**d**) 4%; (**e**) 5%; and (**f**) the relationship between a sample and roughness.

Figure 2 shows the hydrogen-terminated diamonds prepared at different CH_4_ concentrations. The surface roughness and morphology were characterized by atomic force microscopy (AFM). It can be seen from the diagram that when the CH_4_ concentration increases from 1% to 5%, the surface roughness of the sample increases first, then decreases and increases again, and the roughness RMS varies from 3.06 nm to 1.26 nm. When the CH_4_ concentration is 1%, because of the interaction between diamond growth and hydrogen etching, the CH_4_ concentration is low, the growth effect is less than the etching effect, and the etching effect is enhanced. Due to the selective etching of the surface defects and non-defect areas by hydrogen plasma, the surface roughness is large. When the CH_4_ concentration gradually increases to 2%, we find that the roughness is 3.06 nm. This is because the growth effect is enhanced at this concentration, but the selective region etching generated by the hydrogen plasma still exists, resulting in uneven growth heights in different regions of the surface. It can be seen from Figure 2 that when the concentration of CH_4_ is 3% and 4%, the surface roughness decreases continuously. This is because with the increase in CH_4_ concentration, the growth effect and etching effect gradually reach equilibrium. Therefore, the roughness reaches its lowest value at 4% CH_4_, which is 1.26 nm. However, with the continuous increase in CH_4_ concentration, its growth effect is strong, and overgrowth occurs in local areas of the surface, resulting in inconsistent growth thicknesses in different areas of the surface, resulting in an increase in surface roughness [9,25]. Because the carrier mobility is closely related to the defects and roughness of the surface, the less the defects and the smaller the roughness, the better the electrical transmission characteristics of the carriers. Therefore, after epitaxial growth in 4% CH_4_ and hydrogen plasma etching, better surface carrier electrical characteristics can be obtained [22,24].

**Figure 3:**
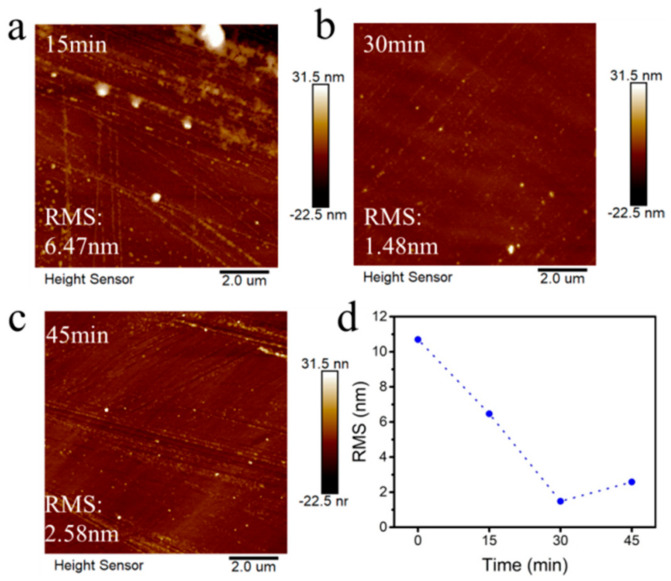
Surface roughness of the homoepitaxial growth layer treated with hydrogen plasma at different times: (**a**) 15 min; (**b**) 30 min; (**c**) 45 min; and (**d**) the relationship between time and roughness.

From Figure 3, it is observed that the surface of the diamond changes from local protrusions to a small number of small protrusions and then to more protrusions as the hydrogen plasma treatment time increases from 0 min to 45 min. When diamond forms a hydrogen terminal, it will etch the surface. Diamond has surface defects, and the etching effect will be enhanced at defects and dislocations during hydrogen plasma hydrogen treatment. It can be observed from the figure that with the extension of hydrogen plasma etching time, the surface roughness of diamond decreases first and then increases. When the etching time is from 15 min to 45 min, the RMS varies from 6.47 nm to 2.47 nm. This is because at 15 min a short period of hydrogen plasma treatment will cause surface growth defects and dislocations to be exposed at the same time, and the roughness is larger. With the hydrogen plasma etching time of 30 min, because the hydrogen plasma etches the edge faster, the steep edges such as steps or deep pits tend to be gentle, resulting in a decrease in roughness. As the hydrogen treatment time is 45 min, the deep pits etched by dislocations and defects lead to an increase in roughness. Since the carrier mobility and surface square resistance are greatly affected by the surface roughness, the smaller the surface roughness, the better the carrier mobility. Therefore, a 30 min hydrogen plasma treatment of epitaxially grown diamond can enhance its carrier mobility.

**Figure 4:**
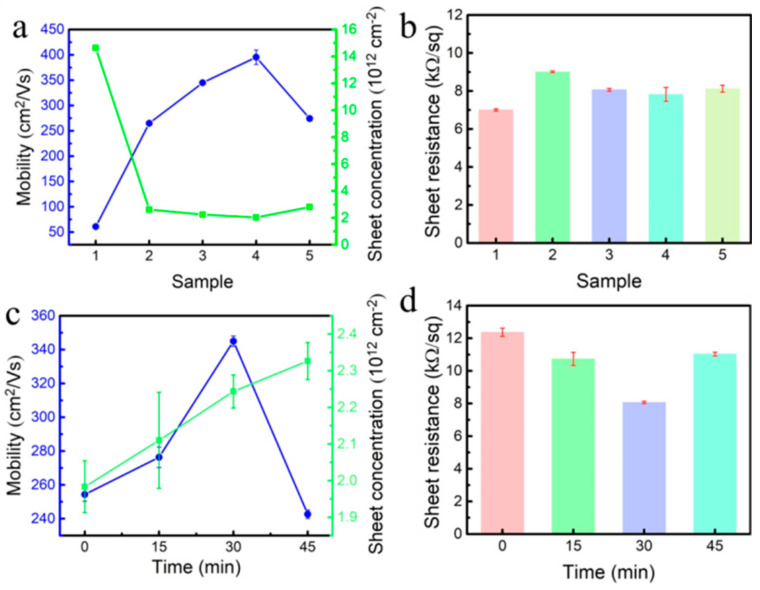
Electrical properties of hydrogen-terminated diamond under different conditions: (**a**) relationship between carrier mobility and carrier concentration and sample; (**b**) relationship between sheet resistance and sample; (**c**) relationship between carrier mobility and carrier concentration and hydrogen plasma treatment time; and (**d**) relationship between sheet resistance and hydrogen plasma treatment time.

Figure 4 shows a hydrogen-terminated diamond prepared under different conditions, and its electrical properties are tested by the Hall effect. From Figure 4a, it can be seen that the carrier mobility of hydrogen-terminated diamond prepared by 1% CH_4_ is 60.8 cm^2^/(Vs), and the carrier concentration is 1.46 × 10^12^/cm^2^. This is because during the diamond growth process, due to the low concentration of CH_4_, the overall growth effect and etching effect are not balanced in the whole growth process [8]. The etching effect is greater than the growth effect, which will reduce the quality of the epitaxially grown diamond, thus affecting the final quality of the hydrogen-terminated diamond. From the Hall effect of the sample, it can be seen that in the process of the CH_4_ concentration increasing from 1% to 5%, the electrical properties of the hydrogen-terminated diamond show a trend of carrier migration first increasing and then decreasing. In sample 4, the carrier mobility reaches a maximum of 395 cm^2^/(Vs). The carrier mobility of the hydrogen-terminated diamond prepared by 5% CH_4_ is 274 cm^2^/(Vs). Compared with the hydrogen-terminated diamond prepared by 4% CH_4_, the carrier mobility of the hydrogen-terminated diamond prepared by 5% CH_4_ decreases sharply. This is due to the excessive growth of the diamond in this state. It is due to the large concentration of CH_4_ in the growth atmosphere, the strong growth effect, and the excessive growth in the local area of the surface, which leads to the uneven surface of the sample and affects the transport of carriers. Figure 4b shows that the square resistance of hydrogen-terminated diamond prepared at different CH_4_ concentrations is not much different. The quality of homogeneous epitaxial diamond films is good, and the impurity content is low, which has little effect on the surface square resistance.

Figure 4c shows the electrical properties of hydrogen-terminated diamond obtained by hydrogen plasma treatment at different times. As the hydrogen plasma treatment time increases from 0 min to 45 min, the carrier mobility increases first and then decreases, and the surface sheet resistance decreases first and then increases. This is because when the surface is etched by hydrogen plasma, the surface will be smoothed, and the surface defects and dislocations will be reduced. When the treatment time is 15 min, the non-diamond phase and surface defects generated by epitaxial growth cannot be completely eliminated. Therefore, compared with the non-hydrogen plasma treatment, the electrical performance is only improved. When the treatment time is 30 min, the surface defects and non-diamond phases can be uniformly reduced, and the electrical properties can be greatly improved. When the treatment time is 45 min, the hydrogen plasma etching effect is enhanced, and the surface is subjected to secondary damage. Defects such as etching pits increase the roughness of the surface, resulting in a weakening of its electrical properties. Therefore, when the hydrogen plasma treatment time is 30 min the surface sheet resistance of the sample is the lowest, which is 8 kΩ/square, and the carrier mobility is the highest, reaching 345 cm^2^/(Vs), and the carrier concentration is 2.24 × 10^12^/cm^2^.

**Figure 5:**
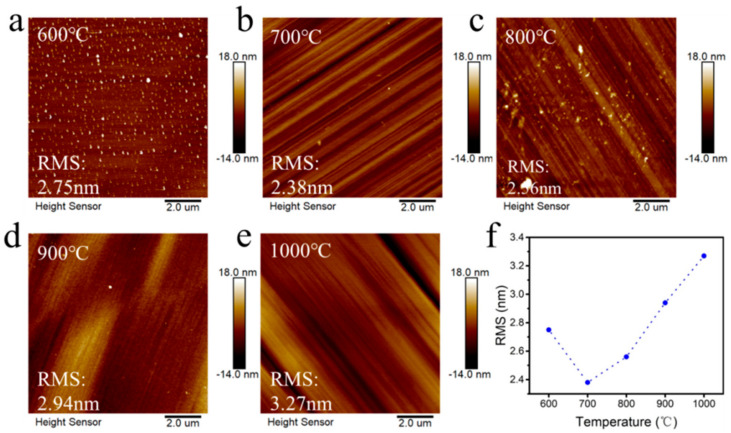
Surface roughness of hydrogen-terminated diamond at different hydrogen plasma treatment temperatures: (**a**) 600 °C; (**b**) 700 °C; (**c**) 800 °C; (**d**) 900 °C; (**e**) 1000 °C; and (**f**) the relationship between temperature and roughness.

As shown in Figure 5, diamond was hydrogen-etched at different temperatures, and its surface roughness was characterized by AFM. It is observed from the diagram that with the increase in hydrogen plasma etching temperature, the RMS of diamond surface roughness decreases first and then increases. When the temperature gradually increases from 600 °C to 1000 °C, the RMS varies from 2.38 nm to 3.27 nm. With the increase in hydrogen plasma treatment temperature, the surface of the diamond shows a different morphology, which is due to the formation of a C-H bond on the surface of the diamond and the etching of the diamond surface by hydrogen plasma. Under 600 °C hydrogen treatment, the surface shows uniform protrusions, the energy density is low, and the removal effect of the defects and scratches on the diamond surface itself is poor. With the increase in hydrogen treatment temperature, there is an obvious etching phenomenon. There are dislocations and defects on the surface of the diamond. Etching pits will be formed at defects and dislocations during hydrogen plasma treatment. Etching pits will increase due to defects, dislocations, and etching, resulting in an increase in the roughness RMS. At the temperature of 800 °C to 1000 °C, the roughness shows a positive growth, which is because the energy density is too high, and the damage of the hydrogen plasma to the surface increases. At 900 °C and 1000 °C, the surface of the material has a significant impurity removal effect, which also improves the purity of the material surface [26] Therefore, hydrogen plasma treatment at 900 °C can make the surface roughness and surface purity reach a better state.

**Figure 6:**
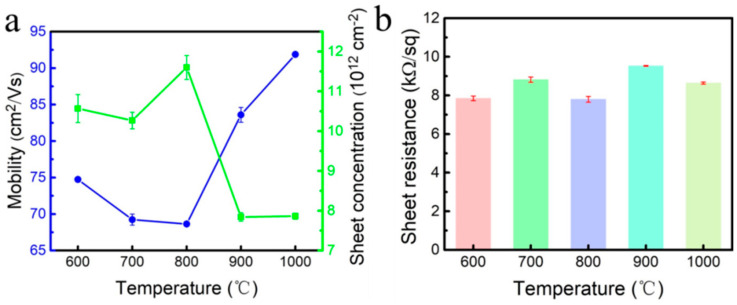
Electrical properties of hydrogen-terminated diamond with different hydrogen plasma treatment temperatures: (**a**) carrier mobility and carrier concentration; (**b**) sheet resistance.

Figure 6 shows hydrogen-terminated diamond prepared by hydrogen plasma treatment on the surface of a diamond substrate at different temperatures. The electrical properties of hydrogen-terminated diamond were tested by the Hall test system. Figure 6a shows that as the hydrogen etching temperature gradually increases from 600 °C to 1000 °C, the carrier concentration increases first and then decreases. This is due to the fact that the energy density gradually increases with the increase in temperature, which makes the surface hydrogenation rate of hydrogen-terminated diamond increase. However, if the temperature is too high, the surface will be damaged, so the carrier concentration decreases [27]. The carrier mobility is related to surface defects and carrier concentration. When the temperature is 900 °C, the mobility is 83.6 cm^2^/(Vs), which is 21.8%-higher than the mobility of 68.4 cm^2^/(Vs) at 800 °C. At the temperature of 1000 °C, the mobility is 91.8 cm^2^/(Vs), which is only 9.8%-higher than the mobility of 900 °C [28]. Therefore, increasing the mobility under relatively mild conditions is the first choice. Figure 6b shows that the sheet resistance of the hydrogen-terminated diamond prepared at 600 °C to 1000 °C is in the range of 7.64 to 8.72 kΩ, and the surface sheet resistance changes little [26]. This is because after hydrogen plasma etching, the impurity content on the surface of the sample is low, the RMS range is 2.38 nm to 3.82 nm, and the surface roughness is not large, so the sheet resistance is not much different.

**Figure 7:**
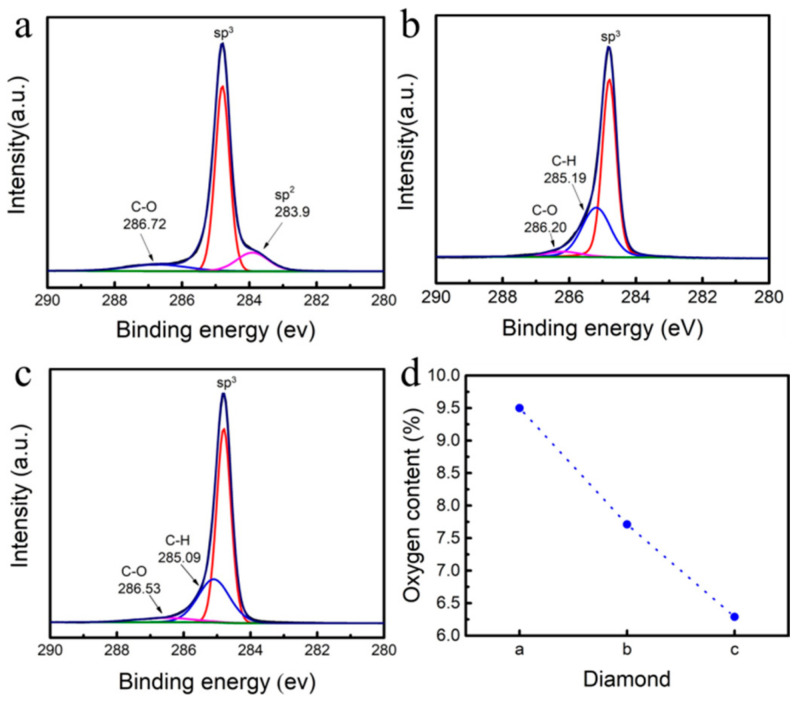
C 1s spectra and oxygen content of different diamonds: (**a**) diamond substrate; (**b**) hydrogen-terminated diamond prepared by hydrogen plasma treatment at 900 °C; (**c**) hydrogen-terminated diamond prepared by homoepitaxial growth of 4% CH_4_; and (**d**) the relationship between three different diamonds and surface oxygen content.

Figure 7 shows the surface chemical bond composition distribution of hydrogen-terminated diamond and the substrate diamond prepared by two different methods. Figure 7a shows that there are oxygen elements on the surface of the substrate diamond, forming C-O bonds, and there is a non-diamond phase. The oxygen element on the surface may be mainly derived from the surface oxygen terminal structure formed by pickling and air oxidation. In Figure 7b, after hydrogen plasma treatment at 900 °C for 30 min it can be clearly seen that the sp^2^ phase on the surface of diamond disappears, and the C-H bond is formed on the surface by the conversion of C-C and C-O [29]. Figure 7c shows hydrogen-terminated diamond prepared by homoepitaxial growth of a diamond film with 4% CH_4_. By analyzing the bonding on the surface, it can be obtained that a layer of diamond film is homoepitaxially grown on the substrate diamond and treated with hydrogen plasma. The C-C conversion occurs on the surface after growth, forming a C-H bond, and there is no sp^2^ hybridization of carbon on the surface of the homoepitaxially grown diamond, and a high-quality diamond film is obtained [30,31]. However, there are also C-O bonds on the surface, which may be due to the fact that the prepared hydrogen-terminated diamond is exposed to air, and the adsorption of oxygen-containing functional groups occurs on the surface [27]. From Figure 7d, it can be seen that the surface oxygen content of hydrogen-terminated diamond prepared by homoepitaxial growth is lower than that of the substrate diamond and diamond surface treated with direct hydrogen plasma [32].

Figure 4a shows that the surface carrier mobility of hydrogen-terminated diamond prepared by homoepitaxial growth of a diamond film with 4% CH_4_ is 395 cm^2^/(Vs). In Figure 6a, the surface carrier mobility of the substrate diamond treated with hydrogen plasma at 900 °C is 83.6 cm^2^/(Vs). The difference in mobility between the two may be due to the existence of the sp^2^ phase affecting the carrier characteristics. A high-quality diamond surface is obtained by epitaxial growth, which reduces the influence of the non-diamond phase and enhances the mobility of carriers. Table 4 shows the comparison of the parameters between this work and other work techniques, and the carrier mobility is much larger than that reported in other works.

## 4. Conclusions

In this paper, the effects of epitaxial growth layer with different CH4 concentrations, hydrogen etching time of epitaxial growth layer and plasma treatment temperature on hydrogen-terminated diamond were studied. When the hydrogen plasma treatment is directly carried out on the diamond substrate, the hydrogen etching temperature is 900 °C, the carrier mobility is lower than that after epitaxial growth, and the sheet resistance is larger, which is related to the surface quality of the diamond substrate. When the hydrogen etching time of the epitaxial growth layer is 30 min, the hydrogen plasma treatment time is the best, and better electrical properties can be obtained. When the concentration of CH_4_ is 4%, the diamond film with a smooth surface and a roughness of 1.26 nm is obtained. The XPS results show that the surface of the diamond film prepared by this process is a hydrogen-terminated diamond structure, and the surface forms a stable C-H bond with P-type conductivity. The Hall test results show that the hydrogen-terminated diamond prepared under this condition has the best electrical properties. The sheet resistance is 7.82 kΩ/square, the carrier mobility is 395 cm^2^/(Vs), and the carrier concentration is 2.03 × 10^12^/cm^2^. The carrier mobility of hydrogen-terminated diamond is significantly improved, which provides a reference for the selection of semiconductor electronic device materials.

## Figures and Tables

**Table 1:** Growth parameters of high-quality diamond homoepitaxial layers.

Sample	CH_4_ Concentration/%	Temperature/°C	Power/W	Pressure/kPa	Duration/h
1,2,3,4,5	1–5	950–980	4200–4600	13–15	8

**Table 2:** Hydrogen plasma treatment parameters.

Temperature/°C	Power/W	Pressure/kPa	Duration/h
600	2600	6.5	0.5
700	3300	9	
800	3800	11	
900	4500	12	
1000	5000	15	

**Table 3:** The growth thickness of the homoepitaxial layer of different samples.

Sample	CH_4_ Concentration/%	Growth Thickness (um)
1	1	4
2	2	5
3	3	12
4	4	18
5	5	28

**Table 4:** Comparison of parameters between this work and other work.

	Carrier Concentration (cm^−2^)	Carrier Mobility (cm^2^/Vs)	Sheet Resistance (kΩ/Square)
**This work**	395	2.03 × 10^12^	7.82
**[33]**	365	2.9 × 10^12^	6
**[34]**	80	3.5 × 10^12^	7
**[35]**	50.5	8.89 × 10^13^	1.388
	57.9	9.81 × 10^13^	1.099

## Data Availability

The data that support the findings of this study are available from the corresponding author upon reasonable request.
